# The incidence, baseline predictors, and outcomes of dementia in an incident cohort of Parkinson’s disease and controls

**DOI:** 10.1007/s00415-022-11058-2

**Published:** 2022-03-21

**Authors:** Carl Counsell, Cinzia Giuntoli, Qaisar Imran Khan, Jodi Maple-Grødem, Angus D. Macleod

**Affiliations:** 1 Institute of Applied Health Sciences, University of Aberdeen, Polwarth Building, Foresterhill, Aberdeen, AB25 2ZD UK; 2 University of Aberdeen Medical School, Foresterhill, Aberdeen, AB25 2ZD UK; 3 The Norwegian Centre for Movement Disorders, Stavanger University Hospital, Stavanger, Norway; 4 Department of Chemistry, Bioscience and Environmental Engineering, University of Stavanger, Stavanger, Norway

**Keywords:** Parkinson’s disease, Dementia, Incidence, Cohort studies, Prediction

## Abstract

**Background:**

There are few long-term data on the incidence, baseline predictors, and outcomes of dementia in Parkinson’s disease (PD) from prospective community-based incident cohorts.

**Methods:**

The PINE study prospectively identified all incident PD patients in Aberdeen along with age–sex-matched, community-based controls who consented to standardized annual life-long follow-up. Each year, a clinical expert reviewed the diagnosis of PD and the presence of dementia according to DSM-IV-based criteria. Age–sex stratified incidence rates for dementia in PD and controls were calculated and compared with hazard ratios (HR) adjusted for age, sex, education, and socioeconomic status. Cox proportional-hazard modelling was used to assess baseline predictors for PD dementia and the influence of dementia on survival and institutionalization.

**Results:**

201 patients (mean age 72.6yrs) and 260 controls (mean age 75.4yrs) were followed for median 9.5 years. The incidence of dementia was 7.4 (PD) versus 2.1 (controls) per 100 person-years (adjusted HR 6.0, 95%CI 4.1–8.7), with a sixfold increase from under 60 to over 80 years in PD but no sex difference. Independent baseline predictors of PD dementia were older age at diagnosis, self-reported cognitive symptoms, dream enactment, lower MMSE scores, worse motor UPDRS scores, and the *ApoE* genotype. PD dementia increased the rates of subsequent death and institutionalization (32.0 and 26.9 per 100 person-years, respectively).

**Conclusion:**

The incidence of dementia in PD is high, increases markedly with age, is increased in those with baseline subjective cognitive symptoms as well as other established risk factors, and is associated with high rates of death and institutionalization.

**Supplementary Information:**

The online version contains supplementary material available at 10.1007/s00415-022-11058-2.

## Introduction

Dementia is one of the key non-motor features of Parkinson’s disease (PD), which is associated with increased mortality [1], poorer quality-of-life [2], worse carer burden [2], and higher rates of institutionalization [3]. A systematic review showed the prevalence of dementia in PD was about 24–31% [4]. However, there are relatively few reliable data on the annual or cumulative incidence of dementia in PD, especially over the long term. Widely varying annual incidence rates (5–11 per 100 person-years) [5] and cumulative proportions (3–28%) have been quoted up to 4 years post-diagnosis [6], whilst at 20 years, 20–80% of PD survivors have been found to have dementia [6, 7]. Some of the differences relate to the nature and age of the cohorts studied, many being selected, largely hospital-based cohorts with relatively young ages of onset, which have limited generalizability. Very few studies consider the incidence of dementia in unselected incident, population-based PD cohorts with prospective long-term follow-up [5, 8–12]. Those that do have mostly reported short-term results with incidence rates between 2 and 6 per 100 patient-years in the first 5 years, rising with duration of follow-up, and a cumulative proportion of about 10% at 3 years rising to 46% at 10 years (Supplementary Table 1). The rates of dementia in PD were over twice that in the general population [10, 12].

The limited reliable data on the incidence of PD dementia (PDD) also mean that there are relatively limited data on the early clinical factors that predict the subsequent development of dementia. Previous studies have suggested numerous factors such older age at onset, male sex, fewer years of education, worse baseline motor and cognitive scores, mild cognitive impairment, REM sleep disturbance, visual hallucinations, orthostatic hypotension, vascular risk factors, obesity and genetic polymorphisms in the apolipoprotein E (*ApoE*), β-glucocerebrosidase (*GBA*), and microtubule-associated protein tau (*MAPT*) gene are associated with subsequent dementia [6, 13–15], but some of these are inconsistent associations. In addition, some risk factors for other dementias such as subjective cognitive decline have not been studied in PDD [6, 16]. Therefore, there is a need for more robust data on early risk factors from unselected population-based cohorts.

We aimed to assess the incidence of PDD by age and sex in a well-characterized, unselected, prospective population-based incident cohort of people with PD compared to a concurrent control group and to identify baseline predictors for PDD and outcomes following the development of PDD.

## Methods

The Parkinsonism Incidence in North-East Scotland (PINE) study used multiple overlapping search strategies to identify all patients with a newly diagnosed degenerative or vascular parkinsonian syndrome from primary care practices (baseline population about 315,000) in and around Aberdeen, Scotland, in two phases (2002–04, 2006–09) [17]. Parkinsonism was defined as two or more cardinal motor signs (bradykinesia, rigidity, rest tremor, otherwise unexplained postural instability). Eligible patients were offered life-long yearly follow-up (still ongoing) by a consultant neurologist with an interest in movement disorders (CC) or a supervised clinical research fellow with linkage to the national death register to establish survival. Clinical care was not altered by participation in the study. For each eligible patient who consented to follow up, we tried to identify an age–sex-matched community-based control without PD by approaching up to four people closest in age to the patient from the same primary care practice or from a register of elderly people who had taken part in a previous community-based screening project [18].

At each annual review, the cause of parkinsonian syndrome was classified using all available information (clinical syndrome, atypical features, response to dopamine replacement therapy, development of motor complications, and results of structural (CT or MRI) or dopamine transporter SPECT imaging where undertaken) using clinical expertise guided by the appropriate research criteria (the UK Brain bank criteria for PD [19]) or from pathology in those who died and had given consent for post-mortem. For the purposes of this report, we only included patients with PD and all controls.

### Assessments

Patients and controls who gave consent had a standardized baseline (diagnostic) visit and yearly review including clinical examination looking for atypical features and assessment of Unified Parkinson’s Disease Rating Scale [UPDRS], disability [Schwab & England (S&E) and Barthel index], cognitive function (mini-mental state examination (MMSE) and mini-mental Parkinson’s [MMP] [20]), mood (Geriatric Depression Scale-15 item version), and a brief questionnaire for non-motor complications including symptoms of REM sleep behaviour disorder (i.e. dream enactment) and subjective cognitive symptoms (“do you feel that your memory or thinking processes are worse than they should be?”). Some patients only consented to limited assessment including UPDRS motor score, S&E score, MMSE, and the checklist of motor and non-motor complications. Patients who consented to all aspects of the study gave blood for genotyping including testing of *MAPT* H1 versus H2 haplotype, *ApoE* ε4 allele, and *GBA* gene variants using previously described methods [21], whereas those who consented to more limited assessment did not have blood taken for genetic testing. Those who were unable to come to clinic were visited at home. Controls were able to consent to remote follow-up (annual medical records review, questionnaires, and linkage to national death register) or in-person review in which case they completed the same assessments as patients, performed by a research nurse.

### Definition of dementia

Dementia in patients and controls was diagnosed clinically according to DSM-IV criteria by an experienced clinician with expertise in diagnosing dementia, namely either the study neurologist or a consultant psychiatrist if the latter had seen the patient as part of their clinical care (identified by screening primary and secondary care medical records for each patient and control). There needed to be evidence of progressive cognitive decline (detected from prospective follow-up), involving at least two cognitive domains (from attention, executive function, visuospatial function, verbal and visual memory, language, agnosia, and apraxia) assessed from the history, examination, and MMSE and MMP cognitive tests, which was limiting daily activities, social or occupational function in the absence of delirium. We did not apply strict cut-off scores from cognitive tests or require formal neuropsychological testing. All PD patients who developed dementia were presumed to have PDD (all met clinical criteria for probable or possible PDD [22]). The date of dementia was defined as the date it was first diagnosed clinically.

### Data analysis and statistical methods

Data were extracted after follow-up until 31st May 2020 and double-checked prior to analysis. Follow-up time was calculated using the reverse Kaplan–Meier method [23]. Incidence rates of dementia were calculated by dividing the number of cases by the total number of person-years at risk, stratified by age and sex. Confidence intervals were calculated assuming a Poisson distribution. Kaplan–Meier curves of time-to-dementia were plotted censoring for last follow-up or death and the hazard ratio (HR) for dementia in patients compared to controls was calculated using Cox proportional-hazards modelling controlling for baseline age, sex, years of education, and socioeconomic status [24]. We tested whether there was a significant interaction between patient/control status and sex. We also did a sensitivity analysis excluding controls who consented to remote rather than in-person follow-up to investigate whether this altered the incidence rate or hazards ratio comparing patients and controls.

Several potential baseline variables that might predict subsequent dementia in PD were identified from the previous literature (supplementary Table 2) and were entered into a Cox proportional-hazards model to identify the statistically significant predictors of PDD. The primary analysis (model 1) included ten baseline variables (measured at, or shortly after, diagnosis) with minimal missing data: age, sex, years of education, smoking history, body mass index, history of vascular disease (defined as history of previous ischemic heart disease, stroke, transient ischemic attack or peripheral vascular disease) or diabetes, presence of subjective cognitive symptoms, history of dream enactment behaviour, MMSE, and UPDRS part 3. The number who developed dementia per variable was just under 10 [25]. Secondary analyses included other variables which were not collected in all patients (due to different levels of study participation). Model 2 also included National Adult Reading Test (NART) score as an alternative measure for premorbid intelligence than years of education [26], GDS-15 as depression measure, and MMP instead of MMSE as a measure more likely to capture cognitive deficits in PD. Model 3 added the three genetic variables to the model 1 variables. In model 1, missing predictor variable data were imputed with multiple imputation, assuming that data were missing at random. We used a predictive mean matching algorithm to impute data using all the predictor variables in the model, the dementia variable, the Nelson–Aalen estimator of the cumulative hazard function, and the year 1 MMSE values (because some baseline MMSE values were missing). For each model, 20 imputed datasets were combined using Rubin’s rules. The missing data in the additional variables included in models 2 and 3 were all missing in most cases with lower levels of study participation, so we only included patients with complete data in these models. We excluded one person with longstanding learning disability but no dementia from the prediction models, because this was unusual clinically and their low baseline scores (MMSE 18/30, MMP 15/32) were highly influential on the modelling.

We investigated the risk of institutionalization and death after dementia. We plotted Kaplan–Meier probabilities of remaining (i) living at home (not in an institution) and (ii) alive after dementia in both PD and controls. Participants were censored at death (in the institutionalization analysis) or when last seen in those with ongoing follow-up. Hazard ratios comparing these outcomes in PD versus controls were calculated using Cox regression adjusted for age at baseline and sex. We investigated the influence of dementia on both these outcomes in time-dependent Cox regression models with the development of dementia as a time-varying covariate (i.e., dementia coded as 0 until time of development of dementia and as 1 thereafter). These models were adjusted for baseline age, sex, and the other significant predictors of dementia in Model 1. The proportional-hazards assumption was verified by visual inspection of log–log plots and a formal test based on Schoenfeld residuals (the estat phtest command in Stata). Functional form was assessed with fractional polynomials [27]. Data were analysed using Stata version 16.1.

## Results

**Fig. 1:**
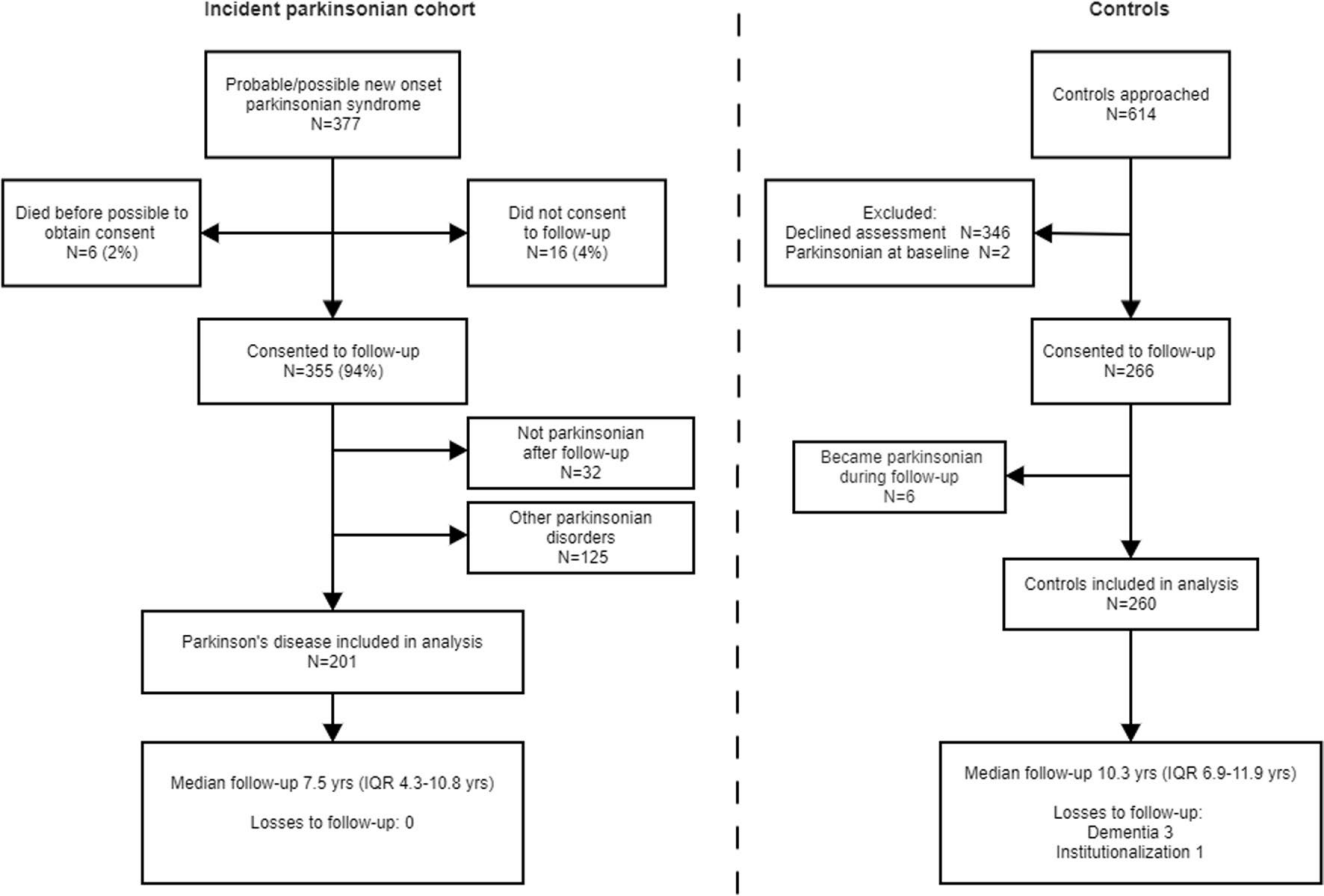
PINE study flowchart

The recruitment, follow-up, and baseline characteristics of 201 patients and 260 controls are shown in Fig. 1 and Table 1. Four people with PD (with delayed diagnosis from motor onset) had dementia at baseline assessment and were excluded from all survival analyses. Controls were slightly older than patients as we included all controls, not just those age-matched to people with PD.

**Table 1:** Baseline characteristics of patients and controls

	Parkinson’s disease				Controls			
	N missing	No dementia *N* = 104	Dementia *N* = 97	All *N* = 201	N missing	No dementia *N* = 214	Dementia *N* = 46	All *N* = 260
Mean age at baseline (SD)	0	70.4 (12.0)	75.0 (7.5)	72.6 (10.3)	0	74.6 (9.8)	79.3 (5.4)	75.4 (9.3)
Male sex	0	67 (64%)	56 (58%)	123 (61%)	0	136 (64%)	22 (48%)	160 (62%)
DepCat 1–3	0	61 (59%)	67 (69%)	128 (64%)	0	117 (55%)	25 (54%)	142 (55%)
History of vascular disease or diabetes	0	35 (34%)	31 (32%)	66 (33%)	0	66 (31%)	15 (33%)	81 (31%)
Median years of education (IQR)	4	11 (11–15)	11 (10–14)	11 (10–14)	40	11 (10–13)	11 (10–13)	11 (10–13)
Median motor UPDRS (part III) (IQR)	0	22 (14–33)	26 (19–32)	22 (14–33)	16	2 (0–4)	4 (2–7)	2 (0–5)
Median GDS-15 (IQR)	38	3 (2–6)	3 (2–5)	3 (2–6)	19	1 (0–2)	2 (1–3)	1 (0–3)
Median MMSE (IQR)	15	29 (28–30)	28 (27–29)	29 (28–30)	18	29 (28–30)	28 (27–29)	29 (28–30)
Median MMP (IQR)	38	30 (28–31)	28 (26–30)	29 (27–30)	17	30 (28–31)	29 (27–30)	30 (28–31)
Subjective cognitive symptoms	0	13 (13)	25 (26)	38 (19)	15	12 (6)	11 (27)	23 (9)
Median NART score (IQR)	76	33 (28–40)	34 (25–40)	33 (27–40)	75	35 (28–41)	32 (24–39)	35 (27–40)
Dream enactment symptoms	0	10 (10)	23 (25)	33 (16)	15	12 (6)	2 (5)	14 (6)
Smoking	1				0			
Current		40 (38)	39 (41)	79 (40)		99 (46)	31 (67)	130 (50)
Ex		7 (7)	5 (5)	12 (6)		24 (11)	3 (7)	27 (10)
Never		57 (55)	52 (54)	109 (55)		91 (43)	12 (26)	103 (40)
Mean BMI (SD)	25	25.9 (4.3)	26.2 (3.8)	26.0 (4.1)	26	27.9 (4.7)	26.2 (3.8)	27.6 (4.6)
*ApoE* ε4 carrier	56^a^	20 (27)	26 (37)	46 (32)	260	NA	NA	NA
Any *GBA* variant	58^b^	6 (8)	10 (14)	16 (11)	260	NA	NA	NA
*MAPT* H1/H1	56^a^	52 (69)	48 (69)	100 (69)	260	NA	NA	NA

*ApoE* apolipoprotein E, *BMI* body mass index, *DepCat* deprivation category based on Carstairs index (1–3 is better socioeconomic status), *GBA* glucocerebrosidase, *GDS-15* geriatric depression Scale-15 item, *HR* hazards ratio, *IQR* inter-quartile range, *MAPT* microtubule-associated protein tau, *MMSE* mini-mental state examination, *MMP* mini-mental Parkinson’s, *NA* not available, *NART* national adult reading test, *SD* standard deviation, *UPDRS* unified Parkinson’s disease rating scale

^a^47 patients did not give consent for genetic testing, 9 had no sample or failed DNA extraction

^b^47 patients did not give consent for genetic testing, 9 had no sample or failed DNA extraction, and 2 failed GBA assay

**Fig. 2:**
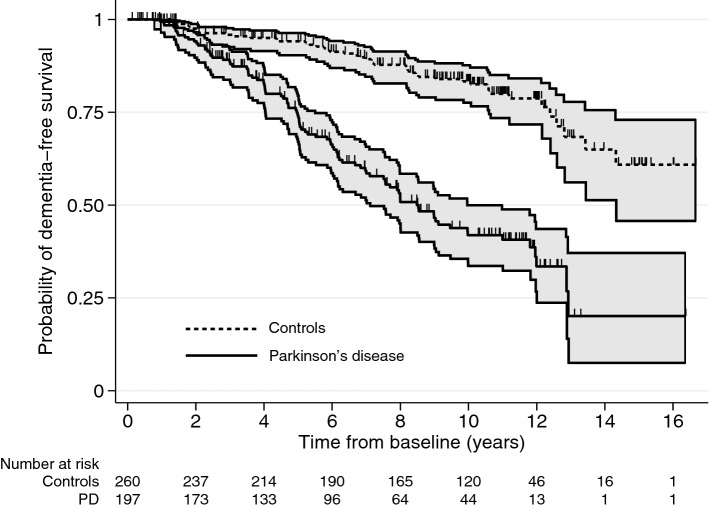
Kaplan–Meier curves for survival free of dementia in incident Parkinson’s disease and controls

Ninety-three patients and 46 controls developed dementia over a median follow-up of 9.5 years (inter-quartile range [IQR] 5.4–11.4). Onset of dementia was at a younger age in PD (Table 1). The overall incidence rate of dementia in PD was 7.4 cases per 100 person-years (95% CI 6.1–9.1) compared to 2.1 cases per 100 person-years (95% CI 1.6–2.8) in controls, but it was significantly dependant on baseline age, especially in PD (1.6 per 100 person-years under 60 versus 12.2 over 80) (Table 2). Kaplan–Meier dementia-free survival curves are shown in Fig. 2 Median time-to-dementia in PD was 8.5 years (95% CI 7.0–11.0) and was not reached in controls. In PD, 25.8% developed dementia by 5 years, 58.1% by 10 years, and 79.9% by 15 years compared to 5.9%, 16.7%, and 39.1%, respectively, in controls.

**Table 2:** Dementia incidence and outcomes after dementia

	Parkinson’s disease		Controls	
	No dementia	Dementia	No dementia	Dementia
	*N* = 104	*N* = 97^a^	*N* = 214	*N* = 46
Dementia incidence (N/person-years = incidence rate/100 person-years (95% confidence interval))				
All		93/1250.5 = 7.4 (6.1–9.1)		46/2226.0 = 2.1 (1.5–2.7)
Men		52/706.4 = 7.4 (5.6–9.7)		22/1399.4 = 1.6 (1.0–2.4)
Women		41/548.4 = 7.5 (5.5–10.1)		24/815.7 = 2.9 (2.0–4.4)
Age < 60		4/252.4 = 1.6 (0.6–4.2)		0/265.4 = 0 (undefined)
Age 60–69		15/263.6 = 5.7 (3.4–9.4)		1/327.8 = 0.3 (0.0–2.2)
Age 70–79		49/533.5 = 9.2 (6.9–12.1)		20/1027.1 = 1.9 (1.3–3.0)
Age 80 +		25/205.2 = 12.2 (8.2–18.0)		25/605.8 = 4.1 (2.8–6.1)
Median age at dementia (IQR)		80.4 (75.6–85.3)		86.8 (83.3–89.4)
Median time in years from baseline to dementia (95% CI)		8.5 (7.0–11.0)		Not reached
Number dead (%)	69 (66)	67 (69)	105 (49)	30 (65)
Median age at death (IQR)	81.1 (76.1–86.5)	84.3 (79.2–88.0)	87.1 (81.8–90.3)	90.1 (87.0–92.8)
Median time in years from baseline to death (95% CI)	7.3 (5.5–8.8)	7.8 (7.0–8.8)	12.5 (10.9–14.8)	10.6 (8.5–12.9)
Median time in years from dementia to death (95% CI)		3.1 (2.3–3.6)		3.1 (2.2–5.8)
Number institutionalized (%)	13 (13)	59 (61)	24 (11)	21 (46)
Median age at institutionalization (IQR)	77.7 (72.1–83.5)	81.2 (77.5–86.7)	92.2 (87.8–94.1)	88.5 (83.4–89.7)
Median time in years from baseline to institutionalization (95% CI)^b^	Not reached	8.0 (6.5–9.0)	15.6 (14.4-undefined)	11.0 (8.9-undefined)
Median time in years from dementia to institutionalization (95% CI)^c^		2.7 (2.1–3.2)		4.3 (1.7-undefined)

All median times in this table were calculated from Kaplan–Meier probabilities

*CI* = confidence interval

^a^4 patients were excluded from survival analyses of dementia, because they developed dementia prior to baseline assessment

^b^1 patient and 2 controls were institutionalized before baseline

^c^20 patients and 4 controls developed dementia after time of institutionalization

**Fig. 3:**
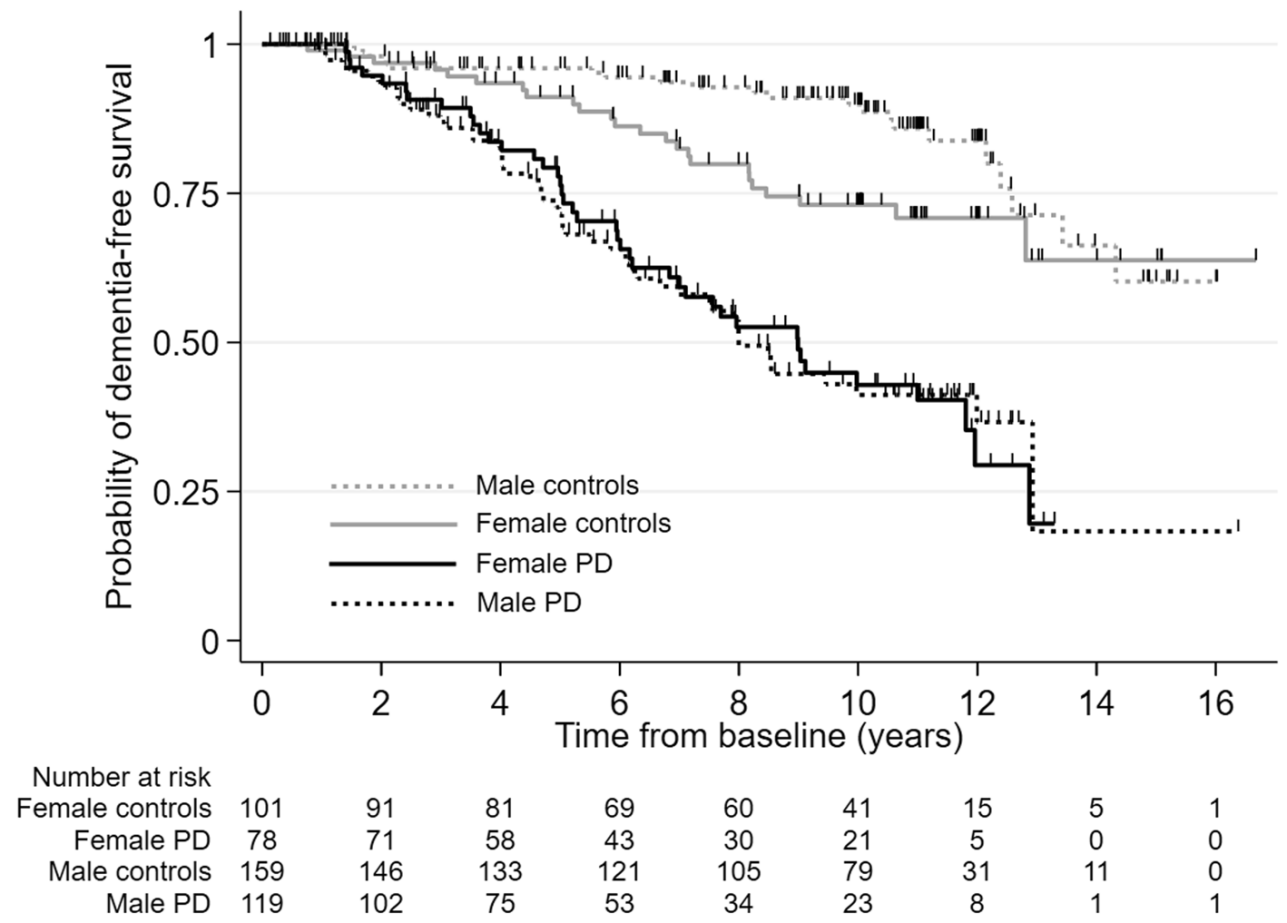
Kaplan–Meier curves for survival free of dementia in incident Parkinson’s disease (PD) and controls by sex

The HR of dementia in PD versus controls adjusted for baseline age, sex, years of education, and socioeconomic deprivation score was 6.0 (95% CI 4.1–8.7), but there was a significant interaction between sex and patient/control status and the risk of dementia (*p* value for interaction was 0.03). There was no sex difference in the risk of dementia in PD, whereas in controls, dementia was more common in women (Fig. 3). Hence, the adjusted HR for dementia was higher in men (8.7, 95% CI 5.2–14.7) than in women (3.9, 95% CI 2.3–6.5). In the sensitivity analysis excluding 18 controls who only consented to remote follow-up, the incidence rate in controls was slightly lower (1.8 cases per 100 person-years) and the adjusted HR comparing PD with controls was slightly higher [6.7 (95% CI 4.5–10.0)] than in the main analyses including all controls.

Significant baseline prognostic factors for PDD in the multivariable analyses were age, presence of subjective cognitive symptoms, history of dream enactment behaviour, motor UPDRS score, MMSE score, and the *ApoE* ε4 allele (Table 3). None of the other prognostic factors studied were significantly associated when adjusted for confounders. The proportional-hazards assumption was satisfied and there was no evidence of non-linearity of interval variables.

**Table 3:** Predictors of Parkinson’s disease dementia

	Baseline variable	Univariable analysis		Multivariable analysis	
		HR (95% CI)	*p* Value	HR (95% CI)	*p* Value
Model 1^a^ (*N* = 196)	Age (10-year increase)	2.17 (1.67–2.81)	< 0.001	2.36 (1.72–3.25)	< 0.001
	Male vs female sex	1.03 (0.68–1.55)	0.91	0.74 (0.48–1.16)	0.19
	Years of education	0.94 (0.87–1.02)	0.14	1.05 (0.97–1.14)	0.23
	Smoking				
	Ex vs never	1.36 (0.54–3.43)	0.51	1.24 (0.47–3.29)	0.67
	Current vs never	1.74 (1.13–2.67)	0.01	1.16 (0.73–1.83)	0.53
	BMI	0.96 (0.91–1.01)	0.12	0.97 (0.90–1.04)	0.36
	History of vascular disease or diabetes	1.28 (0.83–2.00)	0.27	0.71 (0.44–1.15)	0.16
	History of cognitive symptoms	2.31 (1.41–3.78)	0.001	1.98 (1.16–3.40)	0.01
	History of dream enactment symptoms	2.24 (1.38–3.65)	0.001	2.63 (1.54–4.49)	< 0.001
	MMSE	0.85 (0.80–0.91)	< 0.001	0.89 (0.81–0.98)	0.02
	UPDRS part 3 (motor) (10-unit increase)	1.41 (1.19–1.68)	< 0.001	1.01 (0.99–1.03)	0.23
Model 2^b^ (*N* = 128)	NART	0.99 (0.96–1.02)	0.67	1.01 (0.97–1.05)	0.64
	GDS-15	1.02 (0.94–1.09)	0.68	0.98 (0.89–1.08)	0.64
	MMP	0.90 (0.85–0.96)	0.001	0.93 (0.86–1.02)	0.12
Model 3^c^ (*N* = 141)	Any *GBA* variant	1.03 (0.51–2.10)	0.93	1.75 (0.81–3.75)	0.15
	*MAPT* H1/H1 haplotype	0.80 (0.48–1.35)	0.41	0.63 (0.36–1.09)	0.10
	*ApoE* ε4 carrier	1.82 (1.11–2.98)	0.02	3.12 (1.66–5.87)	< 0.001

*ApoE* apolipoprotein E, *BMI* body mass index, *CI* confidence interval, *GBA* glucocerebrosidase, *GDS-15* Geriatric Depression Scale-15 item, *HR* hazards ratio, *MAPT* microtubule-associated protein tau, *MMSE* mini-mental state examination, *MMP* mini-mental Parkinson’s, *NART* National adult reading test, *UPDRS* Unified Parkinson’s Disease Rating Scale

^a^Multivariable analysis adjusted for all other variables listed in model 1

^b^Multivariable analysis adjusted for all variables listed in model 1 except years of education and MMSE

^c^Multivariable analysis adjusted for all variables listed in model 1

**Fig. 4:**
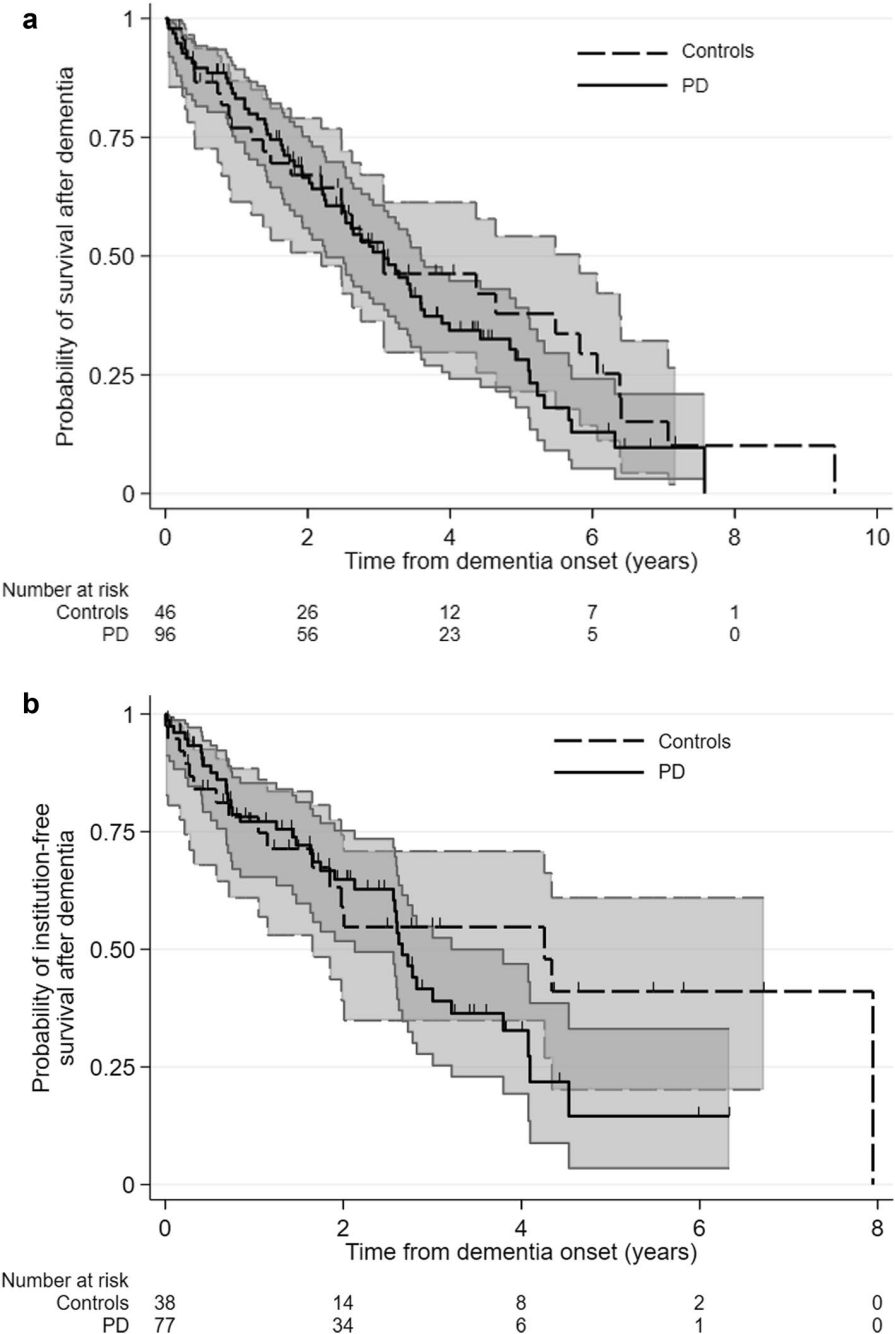
Kaplan–Meier curves for (**a**) death and (**b**) survival free of institutionalization following dementia in PD and controls (shaded areas = 95% confidence intervals)

Kaplan–Meier survival curves of the risk of death and institutionalization after dementia are shown in Fig. 4, but should be interpreted cautiously more than 4 years after dementia onset due to the very small number of surviving patients and controls who were event-free. The rate of institutionalization after dementia was 26.9 per 100 person-years (95% CI 19.6–36.8) in PD and 21.4 per 100 person-years (95% CI 13.3–34.4) in controls (see Table 2). Median survival free from institutionalization after dementia was lower in PD (2.7 years, 95% CI 2.1–3.2) than controls (4.3 years, 95%CI 1.7 to undefined). The mortality rate after dementia was 32.0 per 100 person-years (95% CI 25.9–39.7) in PD and 23.7 per 100 person-years (95% CI 16.5–33.8) in controls. Median survival after dementia was similar in PD and controls: 3.1 years (95%CI 2.3–3.6) in PD and 3.1 years (95% CI 2.2–5.8) in controls. The risk of institutionalization and death after dementia were non-significantly higher in PD than controls (HR 1.8, 95%CI 0.9–3.7 and 1.5, 95% CI 0.9–2.5, respectively), adjusted for baseline age, sex, cognitive symptoms, dream enactment behaviour, and MMSE.

In PD, the development of dementia (as a time-varying covariate in Cox model, adjusted for the same potential confounders) was associated with an increased risk of death (HR 2.5, 95% CI 1.6–3.7) and institutionalization (HR 5.6, 95% CI 3.1–10.0).

## Discussion

We have shown that dementia in PD has a high incidence (7.4% per year), significantly more than in a control population, and had similar rates in men and women unlike controls where it was more common in women, probably because Alzheimer’s is more common in women [28]; came on at a younger age in PD than controls; rose sharply with age at PD diagnosis; and was associated with worse outcomes (annual rates of institutionalization and death 27 and 30 per 100 person-years, respectively), some of which were worse than in controls who developed dementia, probably due to the co-existent motor difficulties of PD. Our incidence rate of PDD was higher than most previous estimates (supplementary Table 1), probably due the higher baseline age and longer follow-up duration. The cumulative proportion of people with PD who had developed dementia up to 15 years after diagnosis was nearly 80%.

We also confirmed some of the previously identified early predictors of PDD [age, dream enactment (a proxy for REM sleep behaviour disturbance), motor and cognitive function, *ApoE* ε4 allele]. However, for the first time, we identified that subjective concern about cognitive symptoms at baseline was also a predictor of subsequent PDD, independent of objective cognitive testing, as described in other dementias [16]. This means that clinicians need to be cautious about dismissing people with Parkinson’s concerns about their memory, even if routinely applied cognitive testing is normal. Interestingly, the baseline MMSE cognitive score was more predictive of subsequent dementia than the more PD-specific MMP, despite concerns that the MMSE does not adequately capture cognitive deficits in PD [29].

This study has several strengths, particularly in its design and length of follow-up. It was based on an incident population-based cohort of people with PD with prospective standardized long-term follow-up with no losses to mortality, dementia, and institutionalization follow-up in patients and very few in controls. Hence, the risk of selection and attrition bias was minimized, and the results should be generalizable to demographically similar populations. There was active surveillance for dementia and the diagnosis of both PD and dementia was made by expert clinicians. PD was confirmed pathologically in 25% of the patients. Consistency of follow-up gave the opportunity to confirm progressive cognitive decline impacting on daily function. It is, therefore, unlikely that cases of clinically significant PDD were missed (unless they developed it in the maximum 1 year period between their last clinical visit and their death) and the diagnosis of PD was as accurate as it could be. However, it is possible that some of the controls who consented to remote notes/questionnaire-based follow-up and were not seen in-person may have developed dementia and been missed, especially if they were not referred for assessment to secondary care by their primary care physician, but the sensitivity analysis excluding these controls did not suggest this was the case.

There are some other weaknesses in the current study. The numbers were relatively small, so the confidence intervals for incidence rates in specific age groups were wide. It also meant we were limited in how many predictive variables we could use in the models to avoid overfitting [25] and there would have been limited power for identifying weaker predictors. We did not assess anticholinergic medication burden as an independent risk factor for PD dementia, because previous work had not shown it to be a predictive factor in our cohort [30]. In addition, we did not have complete baseline data for some predictor variables, again limiting sample size in the secondary analyses. Some potentially important baseline predictors were not available. For example, we did not formally diagnose mild cognitive impairment, a known baseline predictor of PDD [13], although baseline cognition was captured objectively and subjectively. The primary cognitive score was the MMSE which is not recommended for use in PD [29], but the more PD-specific MMP measure was found to be less predictive of dementia. We did not use the Montreal Cognitive Assessment, because this had not been developed when we started the PINE study in 2002. We also did not have the resources to formally diagnose REM sleep behaviour disorder and so a clinical history of dream enactment was used as a proxy. Although the control group was population-based, it is never truly randomly selected, and so, there is a risk it may have been unrepresentative of the general population and may have underestimated the risk of dementia in controls. In terms of generalizability, the PINE study included few people with young-onset PD due to its low incidence and almost all were Caucasian.

This study has several implications. First, in clinical practice, it helps to provide long-term age–sex-specific incidence rates of PDD to guide patient information on prognosis and management. It will also help guide those who plan health care to provide adequate resources in the future for dementia care in PD. Finally, improved understanding of the rates of PDD and its baseline predictors will help researchers design better, adequately powered trials of potential preventative treatments for PDD.

## Supplementary Information

Below is the link to the electronic supplementary material.

Supplementary file1 (PDF 85 KB)

## Data Availability

Anonymised data used for the analyses in this paper are available from the corresponding author.
