# Supplementary material for: The incidence, baseline predictors, and outcomes of dementia in an incident cohort of Parkinson’s disease and controls
Source: J Neurol. 2022 Mar 21;269(8):4288–98. doi: 10.1007/s00415-022-11058-2 (PMC9294013; doi:10.1007/s00415-022-11058-2)
Supplement: Supplementary file 1 — Supplementary file1 (PDF 85 KB) [file 415_2022_11058_MOESM1_ESM.pdf]

Supplementary data

Supplementary Table 1: Dementia annual incidence rates and cumulative proportion who develop dementia in previous PD incident cohorts

			Incidence rate per 100 person-years (cumulative percentage) at different follow-up durations			
Study	Mean age at dx	Mean motor UPDRS at dx	3-4yrs	5 yrs	7 yrs	10 yrs
PARKWEST [8] (n=198)	67.5yr	22.9	2.1 (9%)			
CAMPAIGN [5,9,10] (n=142)	70.2yr	25.5	3.0 (10%)	3.9 (17%)		5.5 (46%) RR 2.6
NYPUM [11] (n=134)	71.3yr	26.0		6.7 (28%)		
PAQUID [12] (n=44)	76.7yr	Not available			7.4 (42%) RR 2.5	

dx – diagnosis; yr - years

RR = relative risk of dementia in PD versus control population

Supplementary Table 2: Potential baseline predictors of PD dementia

Age

Sex

Education/premorbid IQ

Motor UPDRS

Smoking

Obesity

Vascular risks including diabetes mellitus

Postural hypotension

Depression

Rapid eye movement (REM) sleep behaviour disturbance

Visual hallucinations

Objective cognitive status / mild cognitive impairment

Subjective cognitive decline

Genes (glucocerebrosidase, apolipoprotein E, microtubule associated protein tau)
